# Identical Seeding Characteristics and Cryo‐EM Filament Structures in FTLD‐Synuclein and Typical Multiple System Atrophy

**DOI:** 10.1111/nan.70013

**Published:** 2025-03-26

**Authors:** Patrick W. Cullinane, Yang Yang, Viorica Chelban, Yee Yen Goh, Kirsten Ebanks, Toby Curless, Sarah Wrigley, Eduardo de Pablo‐Fernández, Janice Holton, Sew Peak‐Chew, Catarina Franco, Amanda L. Woerman, Henry Houlden, Thomas T. Warner, Sjors H. W. Scheres, Michel Goedert, Zane Jaunmuktane

**Affiliations:** ^1^ Department of Clinical and Movement Neurosciences UCL Queen Square Institute of Neurology London UK; ^2^ Queen Square Brain Bank for Neurological Disorders UCL Queen Square Institute of Neurology London UK; ^3^ Queen Square Movement Disorders Centre UCL Queen Square Institute of Neurology London UK; ^4^ Medical Research Council Laboratory of Molecular Biology Cambridge UK; ^5^ Department of Structural Biology Van Andel Institute Grand Rapids Michigan USA; ^6^ Department of Neuromuscular Diseases UCL Queen Square Institute of Neurology London UK; ^7^ Department of Microbiology, Immunology, and Pathology, Prion Research Center Colorado State University Fort Collins Colorado USA; ^8^ Division of Neuropathology, National Hospital for Neurology and Neurosurgery University College London NHS Foundation Trust London UK

**Keywords:** cell‐based seeding, cryo‐EM, frontotemporal lobar degeneration, multiple system atrophy, α‐synuclein

## Abstract

**Aims:**

The aim of this study is to identify the prevalence of frontotemporal dementia (FTD)/corticobasal syndrome (CBS) in a large cohort of pathologically confirmed cases of multiple system atrophy (MSA) and to determine the α‐synuclein seeding characteristics and electron cryo‐microscopy (cryo‐EM) filament structure in frontotemporal lobar degeneration with MSA‐type α‐synuclein pathology (FTLD‐synuclein).

**Methods:**

The archives of the Queen Square Brain Bank (1989–2023) were searched for histologically confirmed MSA cases, and those with a clinical diagnosis of FTD/CBS were reviewed for pathological features of FTLD‐synuclein. Phosphotungstic acid (PTA)‐precipitated brain homogenates from FTLD‐synuclein, dementia with Lewy bodies (DLB) and G51D *SNCA* synucleinopathy cases were used to seed aggregation in α‐syn140*A53T‐YFP HEK293T cells. The structure of α‐synuclein filaments from an FTLD‐synuclein case was determined by cryo‐EM.

**Results:**

We identified 283 cases of MSA. Four cases had a clinical diagnosis of CBS, one of which met pathological criteria for FTLD‐synuclein. Genetic studies in this case were negative for *SNCA* variants, and PTA‐precipitated brain homogenates seeded abundant cytoplasmic α‐synuclein inclusions that were morphologically indistinguishable from those of typical MSA but distinct from those of G51D *SNCA* and DLB. MSA Type II α‐synuclein filaments were identified by cryo‐EM.

**Conclusions:**

FTD/CBS is rarely associated with MSA pathology. The cell seeding characteristics and cryo‐EM findings support the classification of FTLD‐synuclein as a subtype of MSA, differentiating it from genetic synucleinopathies, such as those with *SNCA* variants G51D and A53E, which have neuropathological features overlapping with MSA and Lewy body diseases. These cases expand the clinicopathological spectrum of MSA and FTLD and have implications for our understanding of selective neuronal vulnerability in MSA and the interpretation of α‐synuclein biomarker studies.

## Graphical abstract

We report the prevalence of corticobasal syndrome in a large neuropathological cohort of multiple system atrophy (MSA) cases and describe the clinical and pathological features of a case of frontotemporal lobar degeneration with α‐synuclein pathology (FTLD‐synuclein). We show that the cell seeding characteristics and α‐synuclein filament structures in a case of FTLD‐synuclein are identical to those of typical MSA. These findings support the pathological classification of FTLD‐synuclein as a subtype of MSA.

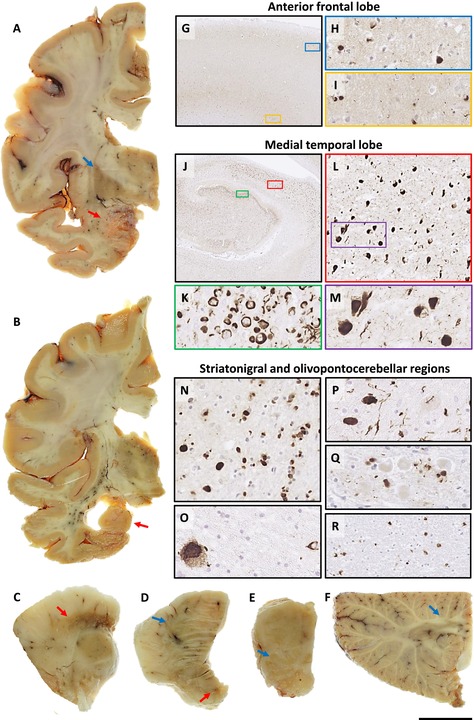

**Abbreviations**
CBD: corticobasal degeneration
CBS: corticobasal syndrome
Cryo‐EM: electron cryo‐microscopy
DLB: dementia with Lewy bodies
DPBS: Dulbecco's phosphate‐buffered sodium
FSC: Fourier shell correlation
FTD: frontotemporal dementia
FTLD: frontotemporal lobar degeneration
FTLD‐synuclein: frontotemporal lobar degeneration with α‐synuclein pathology
GCI: glial cytoplasmic inclusion
MLPA: multiplex ligation‐dependent probe amplification
MND: motor neurone disease
MSA: multiple system atrophy
MSA‐C: multiple system atrophy‐cerebellar subtype
MSA‐MC: multiple system atrophy‐minimal change
MSA‐P: multiple system atrophy‐parkinsonian subtype
NCI: neuronal cytoplasmic inclusion
NFT: neurofibrillary tangle
OPCA: olivopontocerebellar atrophy
PTA: phosphotungstic acid
QSBB: Queen Square Brain Bank for Neurological Disorders
SND: striatonigral degeneration
WES: whole exome sequencing

**Summary**

- Multiple system atrophy (MSA) rarely presents with frontotemporal dementia or corticobasal syndrome.
- This report describes a patient with a final clinical diagnosis of corticobasal syndrome and a disease duration of 8 years.
- Neuropathological examination showed predominantly frontotemporal atrophy with glial cytoplasmic inclusions and heterogeneous neuronal cytoplasmic inclusions in cortical and limbic regions consistent with FTLD‐synuclein.
- Although the neuropathological features of FTLD‐synuclein resemble those of G51D and A53E *SNCA* variant cases, no pathogenic variant in *SNCA* was identified by whole exome sequencing or multiplex ligation‐dependent probe amplification.
- The morphology of seeded inclusions in cultured cells and structures of α‐synuclein filaments derived from this case were identical to those of typical MSA, indicating that FTLD‐synuclein is a rare pathological subtype of MSA.

## 1 Introduction

Multiple system atrophy (MSA) is a sporadic neurodegenerative disease characterised by the accumulation of filaments composed of misfolded α‐synuclein in glial cells (Papp‐Lantos bodies) and in nerve cells [1]. The major neuropathological subtypes are olivopontocerebellar atrophy (OPCA) and striatonigral degeneration (SND), broadly corresponding to the cerebellar (MSA‐C) and parkinsonian (MSA‐P) clinical subtypes [1]. Electron cryo‐microscopy (cryo‐EM) studies have shown that there are two types of α‐synuclein filaments in MSA, each composed of two different protofilaments [2]. They are structurally distinct from the α‐synuclein filaments of Lewy body diseases [3]. Frontotemporal lobar degeneration (FTLD) refers to a group of non‐Alzheimer's neurodegenerative diseases with predominantly frontotemporal atrophy and clinical syndromes that include behavioural‐variant frontotemporal dementia (FTD), primary progressive aphasia, corticobasal syndrome (CBS) and Richardson's syndrome. Protein inclusions associated with FTLD are mainly made of tau, TDP‐43 and FUS‐Ewing sarcoma–TAF15 (FET) [4].

A small number of atypical MSA cases presenting with clinical features of FTD or CBS and FTLD at autopsy have been described [5, 6, 7, 8]. They have been referred to as atypical MSA and FTLD with α‐synuclein pathology (FTLD‐synuclein) and are characterised by (1) varying degrees of FTLD, together with atrophy in striatonigral and/or olivopontocerebellar regions; (2) Gallyas silver‐positive, neuronal cytoplasmic inclusions (NCIs) in cortical and limbic neurones with heterogenous appearance, including Pick body‐like, neurofibrillary tangle (NFT)‐like and ring‐shaped inclusions; and (3) varying amounts of Gallyas silver‐positive α‐synuclein‐immunoreactive glial cytoplasmic inclusions (GCIs).

Unlike typical MSA cases, where pathology is largely restricted to deep cortical laminae, GCIs and NCIs in FTLD‐synuclein cases are also present in the superficial cortical laminae [5]. These features are particularly reminiscent of the characteristics reported for α‐synucleinopathies resulting from variants G51D and A53E in *SNCA* [9, 10, 11, 12], which, in addition to cortical and subcortical white matter GCIs, have prominent neuronal loss and neuronal α‐synuclein pathology in superficial and deep cortical laminae of the frontal and temporal cortices, forming a ‘tramline’ pattern of pathology with similar globular, ring‐shaped and NFT‐like inclusions [10, 11, 12]. Cases with an A53T variant in *SNCA* presenting with FTD and frontotemporal and parietal atrophy on MRI have also been reported [13]. Despite these striking similarities, *SNCA* variants have not been identified in FTLD‐synuclein. The molecular mechanisms responsible for the unusual distribution of pathology and heterogeneity of cellular inclusions in FTLD‐synuclein have not been identified, but the possibility of distinct α‐synuclein strains has been suggested [14]. Here, we report the prevalence of FTD and CBS in a large neuropathological cohort of MSA cases and describe the clinical and pathological features of a case of FTLD‐synuclein. We also describe the morphologies of α‐synuclein aggregates seeded in HEK293T cells using brain homogenates from this case and the cryo‐EM structure of α‐synuclein filaments extracted from the frontal cortex.

## 2 Materials and Methods

### 2.1 Cases

The clinical diagnoses of patients with a pathological diagnosis of MSA referred to the Queen Square Brain Bank for Neurological Disorders (QSBB) between 1989 and 2023 were reviewed to identify cases with a clinical diagnosis of FTD or CBS/corticobasal degeneration (CBD). The primary and secondary care records of identified cases were then reviewed by a neurologist with expertise in movement disorders. All patients had been assessed by hospital specialists (consultant physicians, geriatricians and neurologists).

### 2.2 Neuropathological Examination

Pathological examination of all brains was performed following standard QSBB protocols. For histological analysis, 8‐μm‐thick sections were cut from formalin‐fixed paraffin‐embedded tissue blocks sampled from representative brain regions. Immunohistochemical staining for α‐synuclein (MA1‐90342; Thermo Scientific; 1:1500), amyloid‐β (M0872; Dako; 1:100), phosphorylated tau (MN1020; Thermo Scientific; 1:600) and TDP‐43 (2E2‐D3, H00023435‐M01, Abnova, 1:500) was performed on a Menarini automated staining platform following the manufacturer's guidelines. Biotinylated secondary antibodies were used with horseradish peroxidase‐conjugated streptavidin complex and diaminobenzidine as the chromogen. The sections were counterstained with haematoxylin. MSA cases were classified into one of five pathological subtypes: MSA‐SND, MSA‐OPCA, MSA with equal involvement of SND and OPCA regions (MSA‐SND=OPCA), minimal change MSA (MSA‐MC) and FTLD‐synuclein, based on previously published criteria [5, 15, 16]. The histology slides from the FTLD‐synuclein case (left half brain) were digitised using a digital slide scanner at ×40 magnification (NanoZoomer S360, Hamamatsu), and representative images were taken using the NZConnect (Hamamatsu) slide viewing platform.

### 2.3 Genetics

Whole exome sequencing (WES) was performed on DNA extracted from the cerebellum of the FTLD‐synuclein case. An Illumina HiSeq2500 sequencer was used to generate 100 bp or 150 bp paired‐end reads. Alignment was performed using Burrows‐Wheeler aligner [17] with genome reference consortium human build 38 as a reference. Variants were called using the GATK Unified Genotyper‐based pipeline workflow [18, 19, 20, 21]. All variants were annotated using ANNOVAR [22] and filtered using custom R scripts. Only novel or very rare variants with a minor allele frequency of <0.01 in the genome Aggregation Database v2.1 (gnomAD) [23] were included. Variants were filtered for highly deleterious, rare variants previously associated with monogenic diseases. To investigate for large deletions or duplications, multiplex ligation‐dependent probe amplification (MLPA) of 6 PD‐related genes (*ATP13A2*, *PARK2*, *PARK7*, *PINK1*, *SNCA*, *LRRK2*) was also performed using an MLPA Probemix kit (P051‐D1, MRC‐Holland).

### 2.4 Phosphotungstic Acid Precipitation, Cell Culture and α‐Synuclein Seeding Assay

Phosphotungstic acid (PTA) precipitation was used to extract α‐synuclein aggregates from frozen brain tissues for cell seeding experiments [24, 25]. Frontal cortex, frontal white matter and putamen from the right half of the brain were used for cases of FTLD‐synuclein, G51D *SNCA*, typical MSA and controls. For cases of dementia with Lewy bodies (DLB), the amygdala was used, given the high density of Lewy pathology. Frozen tissue samples were weighed, transferred to Precellys CKMix tissue homogenising tubes (Bertin Technologies) and homogenised in Dulbecco's phosphate‐buffered sodium (DPBS, Thermo Fisher Scientific) using a Precellys 24 homogeniser (Bertin Technologies). Five hundred microlitres of the resulting 10% tissue homogenate (weight/volume) was transferred to a fresh tube and incubated with 75 units of benzonase (Merck) and sarkosyl (Merck) to a final concentration of 2% sarkosyl (weight/volume) for 2 h at 37°C with constant shaking in a thermomixer at 1300 rpm. A 10% (weight/volume) solution of PTA (Merck) dissolved in MilliQ water (adjusted to pH 7.0) was added to the resulting mixture to a final concentration of 2% PTA (weight/volume) and incubated for 16 h at 37°C with constant shaking in a thermomixer at 1300 rpm. The samples were then centrifuged at 16,000 g at 22°C for 30 min. The supernatants were removed and the pellets were resuspended in 2% sarkosyl, before the addition of 10% PTA solution to a final concentration of 2%. The samples were then incubated at 37°C for 1 h with shaking at 1300 rpm, before centrifugation at 16,000 g at 22°C for 30 min. The supernatants were removed, and the pellets were resuspended in 50 μL of DPBS. To prepare the transfection mixture, 2 μL of each resuspended pellet was diluted in 18 μL of DPBS and incubated with 2.5 μL of lipofectamine 2000 (Thermo Fisher Scientific) for 90 min at room temperature before adding 77.5 μL of warm Opti‐MEM (Thermo Fisher Scientific). α‐syn140*A53T‐YFP HEK293T cells were cultured and plated in Dulbecco's modified Eagle medium (Thermo Fisher Scientific) supplemented with 10% (v/v) foetal bovine serum (Thermo Fisher Scientific), 50 units/mL penicillin and 50 μg/mL streptomycin (Thermo Fisher Scientific) and maintained in a humidified atmosphere of 95% O_2_/5% CO_2_ at 37 °C. Cells were counted using a Countess automated cell counter (Thermo Fisher Scientific) before being plated in a 384‐well PhenoPlate (Perkin Elmer) at a density of 4000 cells per well with 0.012 μg Hoechst 33342 (Thermo Fisher Scientific) and incubated for 2–4 h to allow the cells to adhere to the plate. Ten microlitres of the transfection mixture was then added to each of the 6 replicate wells. The plates were returned to the incubator for 5 days, before being imaged using an Opera Phenix high‐content microscope (Perkin Elmer) in the Hoechst 33342 and FITC channels at x20 magnification. Representative images were taken using Columbus image analysis software (Perkin Elmer).

### 2.5 Extraction of α‐Synuclein Filaments and Cryo‐EM Structure Determination

Sarkosyl‐insoluble material was extracted from fresh‐frozen frontal cortex and subcortical white matter, essentially as described [3]. Tissues were homogenised in 20 vol (v/w) extraction buffer consisting of 10 mM Tris–HCl, pH 7.5, 0.8 M NaCl, 10% sucrose and 1 mM EGTA, brought to 2% sarkosyl and incubated for 60 min at 37°C. Following a 10‐min centrifugation at 10,000 g, the supernatants were spun at 166,000 g for 30 min. Sarkosyl‐insoluble pellets were resuspended in 100 μL/g of 20 mM Tris–HCl, pH 7.4, centrifuged at 3000 g for 3 min and applied to glow‐discharged holey carbon gold grids (Quantifoil Au R1.2/1.3, 300 mesh). Glow‐discharging was performed with an Edwards (S150B) sputter coater at 30 mA for 30 s. Aliquots of 3 μL were applied to the grids and blotted with filter paper (Whatman) at 100% humidity and 4°C, using a Vitrobot Mark IV (Thermo Fisher Scientific). Datasets were acquired on a Titan Krios G2 microscope (Thermo Fisher Scientific) operated at 300 kV. Images were acquired using a Gatan K3 detector in super‐resolution counting mode, using a Bio‐quantum energy filter (Gatan) with a slit width of 20 eV. Images were recorded with a total dose of 40 electrons per A^2^. See Table S1 for details.

Movie frames were gain‐corrected, aligned, dose‐weighted and then summed into a single micrograph using RELION's motion correction programme [26]. Contrast transfer function (CTF) parameters were estimated using CTFFIND‐4.1 [27]. All subsequent image‐processing steps were performed using helical reconstruction methods in RELION [28, 29]. α‐Synuclein filaments were picked manually, and reference‐free 2D classification was performed to select suitable segments for further processing. Initial 3D reference models were generated de novo from 2D class averages using an estimated rise of 4.75 Å [30]. Combinations of 3D auto‐refinements and 3D classifications were used to select the best segments for each structure. For all datasets, Bayesian polishing and CTF refinement [31] were performed to further increase the resolution of the reconstructions. Final reconstructions were sharpened using standard post‐processing procedures in RELION, and overall final resolutions were estimated from Fourier shell correlations (FSCs) at 0.143 between the independently refined half‐maps, using phase randomisation to correct for convolution effects of a generous, soft‐edged solvent mask [32]. Atomic model PDB‐ID 6XYP was rigid body‐fitted inside the cryo‐EM map and checked manually in Coot [33]. Coordinate refinements were performed using Servalcat [34] and REFMAC5 [35, 36]. Models were validated with MolProbity [37]. Figures were prepared with ChimeraX [38]. Model statistics are given in Table S1.

### 2.6 Mass Spectrometric Analysis of Sarkosyl‐Insoluble α‐Synuclein

Sarkosyl‐insoluble pellets were resuspended in 100 μL of hexafluoroisopropanol (HFIP). Following a 3 min sonication at 50% amplitude (QSonica), they were incubated at 37°C for 2 h and centrifuged at 100,000 g for 15 min, before being dried by vacuum centrifugation. The pellets were resuspended in 4 M of urea and ammonium bicarbonate (ambic) before being reduced with 5 mM of dithiothreitol at 37°C for 40 min and alkylated in the dark at room temperature for 30 min with 10 mM of iodoacetamide. LysC (Promega) was then added to the samples and digested for 4 h at 25°C. They were diluted to 1.5 M urea with 50 mM of ambic and incubated with another aliquot of LysC (Promega) at 30°C overnight. Digestion was stopped by the addition of formic acid to 0.5%, followed by centrifugation at 16,000 g for 5 min. The supernatants were desalted using C18 stage tips (3 M Empore) packed with Poros oligo R3 (Thermo Fisher Scientific) that were made in‐house. Bound peptides were eluted stepwise with 30%, 50% and 80% acetonitrile in 0.5% formic acid and partially dried in a SpeedVac (Savant). The peptide mixtures were analysed by LC–MS/MS using a Q Exactive Plus hybrid quadrupole‐Orbitrap mass spectrometer, coupled to an Ultimate 3000 RSLC Nanosystem (Thermo Fisher Scientific). The peptides were trapped by a PepMap Neo C18 5 μm 0.3 × 5 mm nano trap column (Thermo Fisher Scientific) and an Aurora Ultimate TS 75 μm × 25 cm × 1.7 μm C18 column (IonOpticks) using solvents consisting of buffer A (0.1% formic acid) and buffer B (80% acetonitrile, 0.1% formic acid) at a flow rate of 300 nL/min. The collected data were analysed using Mascot (Matrix Science, London, version 2.8.2). MS/MS spectra were searched against the Uniprot *Homo sapiens* review fasta database (downloaded May 2023), supplemented with α‐synuclein WT, A53T and G51D sequences. Carbamidomethylation of cysteines was set as a fixed modification, while methionine oxidation and N‐terminal protein acetylation were considered to be variable. Enzymatic specificity of LysC for up to two missed cleavages was permitted. Scaffold software (Proteome Software, version 4.8.4) was used to validate MS/MS spectra. MS/MS spectra of α‐synuclein were selected and manually checked.

## 3 Results

### 3.1 Cases of MSA

We identified 283 cases with a neuropathological diagnosis of MSA, comprising 107 cases of MSA‐SND, 80 cases of MSA‐OPCA, 86 cases of MSA‐SND=OPCA, 7 cases of MSA‐MC and a single case of FTLD‐synuclein. The pathological subtype was not available in two cases. Four previously reported cases (1.4%) had a clinical diagnosis of either CBS or CBS/MSA overlap syndrome^1,39^. The pathological subtypes found in these four cases were MSA‐SND (0.9% of SND cases), MSA‐SND=OPCA (1.2% of SND=OPCA cases), MSA‐MC (14% of MC cases) and FTLD‐synuclein. The clinicopathological and molecular characteristics of the FTLD‐synuclein case are described below. The neuropathological picture suggested that this case may have a G51D variant in the *SNCA* gene.

### 3.2 The Case of FTLD‐Synuclein

This female patient developed short‐term memory impairment and spatial disorientation aged 58. Restless legs syndrome may have begun 14 years earlier, and there was urge incontinence on a background of a hysterectomy performed around 20 years earlier for menorrhagia, with subsequent bladder repair surgery. The patient was not taking regular medication. Her mother developed dementia aged 93 and died aged 97. Her father died in his seventies from ischaemic heart disease. She had three sisters, and two of whom were twins. One of her twin sisters was diagnosed with motor neurone disease (MND) and died aged 71. The patient's cognitive symptoms developed progressively, and she was diagnosed with Alzheimer's disease approximately 5 years after symptom onset. She had several falls over the following year and reported weakness with an inability to fully use her left hand and left‐sided stiffness. There were no visual hallucinations. Examination revealed hypomimia, mild left upper limb tremor and rigidity, weakness of left finger extension and a shuffling gait. She scored 27/30 on a Mini‐Mental State Examination. MRI showed extensive medial temporal lobe atrophy, which was more pronounced on the right. Left‐sided parkinsonism became more prominent over the next 4 months, and the patient had an abnormal dopamine transporter scan. There was worsening urinary incontinence that did not respond to intravesical botulinum toxin injections, and a suprapubic catheter was sited. There was no benefit from levodopa/benserazide (400/100 mg daily); 7 years into the illness, the patient had marked amnestic symptoms, labile mood, depression, sialorrhoea, stimulus‐sensitive myoclonus, progressive dystonia and contractures of the right hand requiring tendon release surgery. The clinical diagnosis was revised to CBS. The patient also developed dysphagia, and a significant orthostatic drop in blood pressure was recorded. During the final year of her life, she developed anterocollis, and her speech was limited to a few words. She died aged 66 after a disease duration of approximately 8 years.

### 3.3 Neuropathology

**FIGURE 1:**
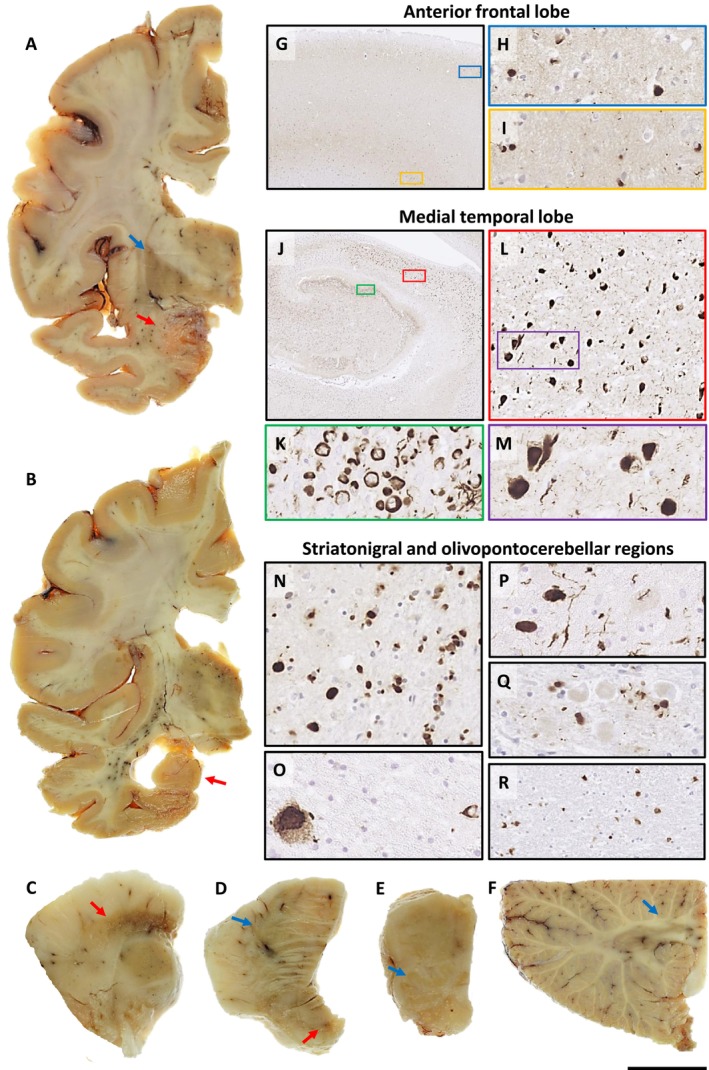
Macroscopic and microscopic findings in the FTLD‐synuclein case. There is prominent atrophy of the medial temporal lobe structures, including the amygdala (A, red arrow), hippocampus and parahippocampal gyrus (B, red arrow). Mild frontal atrophy, enlarged insular space and moderate dilatation of the frontal and temporal horns of the left lateral ventricle can also be seen (A, B). There is a mild reduction in the size of the putamen but without marked lateral atrophy and discolouration (A, blue arrow). There is severe pallor and discolouration of the substantia nigra (C, red arrow). The pontine base (D, blue arrow) shows no significant atrophy, but the locus coeruleus is pale (red arrow). The medulla, including the inferior olivary nucleus (E, blue arrow), the cerebellar cortex and underlying white matter (F, blue arrow), and the dentate nucleus are all well preserved. Abundant NCIs and GCIs are seen in the frontal cortex (G), involving both the superficial (H) and deep (I) cortical laminae with relative sparing of intermediate layers (G). Severe NCI and GCI pathology is seen throughout the hippocampus (J), including abundant ring‐like NCIs in CA1, CA4 and dentate gyrus, where they are seen against a dense meshwork of α‐synuclein‐positive threads (K). Layer CA1 contains numerous NCIs (L), including globular, Pick body‐like NCIs (M). Abundant NCI and GCI pathology is seen in the putamen (N) and substantia nigra (O). Frequent NCIs, GCIs and neuronal intranuclear inclusions are also present in pontine base nuclei against a background mesh of thread pathology (P, Q). Abundant GCIs are also seen in the inferior olivary nucleus (not shown) and cerebellar white matter (R). Scale bar: 1.9 cm in A, B and F; 9 mm in C and E; 9.5 mm in D; 1.4 mm in G; 100 μm in H, I and M; 3.1 mm in J; 80 μm in K; 210 μm in L; 65 μm in N, O and P; 140 μm in Q.

Cerebral atrophy was most prominent in the anterior and medial temporal lobes (Figure 1). There was also mild frontal atrophy. The frontal and temporal horns of the left lateral ventricle were moderately dilated. There was thinning of the cortical ribbon, which was most apparent in the temporal lobe, including parahippocampal and fusiform gyri, and in the anterior orbitofrontal cortex. The caudate nucleus, globus pallidus and thalamus showed mild reductions in size. The putamen was also reduced in size but without the marked lateral atrophy and discolouration that are characteristic of MSA‐SND. The amygdala and hippocampus were severely reduced in bulk. There was severe pallor and discolouration of the substantia nigra and locus coeruleus. The pontine base did not show significant atrophy. The medulla, cerebellar cortex, cerebellar white matter and dentate nucleus were macroscopically normal. Microscopic examination showed severe neuronal loss in the CA1 region of the hippocampus and moderate loss in CA4 and the amygdala. Severe neuronal loss and spongiform changes were also seen in the superficial cortical laminae of the parahippocampal and fusiform gyri. There was neuronal loss and astrogliosis in the lateral putamen and globus pallidus. Pigmented nerve cells were severely depleted in substantia nigra and locus coeruleus. The pontocerebellar fibres and nerve cell populations in the pontine base and inferior olive were well preserved. There was mild patchy loss of Purkinje cells, but the cerebellar white matter showed no apparent gliosis, and the dentate nucleus was unremarkable. α‐Synuclein immunohistochemistry (Figure 1) in the frontal, temporal, parietal and cingulate lobes showed abundant NCIs and GCIs in both superficial and deep cortical laminae, with relative sparing of the intermediate laminae. Numerous GCIs were also seen in the corresponding subcortical white matter. There were many ring‐like NCIs in CA1, CA4 and dentate gyrus, where they were seen against a dense meshwork of α‐synuclein‐positive threads. CA1 also contained many globular, Pick‐body‐like NCIs. Frequent ring‐like NCIs were seen in the superficial cortical laminae of the parahippocampal and fusiform gyri, with many Pick body‐like NCIs in the deeper cortical laminae. There were abundant GCIs in the hippocampus, parahippocampal and fusiform gyri, caudate/putamen and internal capsule. Frequent NCIs were present in the substantia nigra and locus coeruleus with moderate numbers of NCIs and threads seen in the inferior olivary nucleus. Frequent neuronal intranuclear inclusions were also seen in pontine base neurones. GCIs were seen throughout the midbrain, pontine base, medulla and cerebellar white matter. There were scattered diffuse amyloid β‐positive plaques in the frontal cortex, but no tau or TDP‐43 inclusions were observed.

### 3.4 Morphology of Seeded Aggregates

**FIGURE 2:**
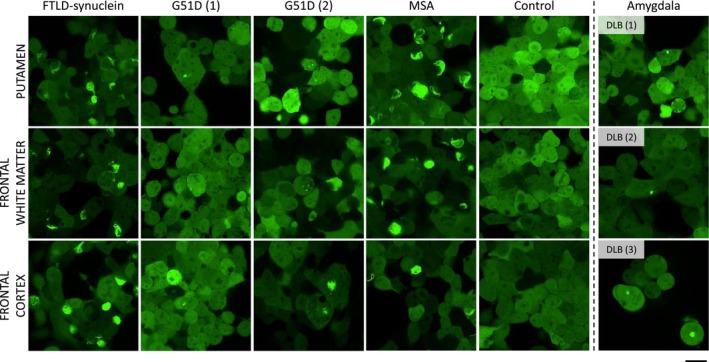
Morphology of seeded aggregates. Representative images of α‐syn140*A53T‐YFP cells transfected with PTA‐precipitated brain homogenates from the putamen, frontal white matter and frontal cortex of FTLD‐synuclein, G51D *SNCA* variant, sporadic MSA and non‐neurodegenerative control cases, as well as from the amygdala of DLB. YFP is shown in green. Scale bar: 20 μm.

PTA‐precipitated brain homogenates derived from the frontal cortex, frontal white matter and putamen of the FTLD‐synuclein case seeded abundant cytoplasmic α‐synuclein inclusions in α‐syn140*A53T‐YFP HEK293T cells. Inclusions ranged from straight or crescent‐shaped threads to tangles of varying sizes that were composed of several elongated threads. The inclusion morphology was indistinguishable from that seen in the same cell line seeded with PTA‐precipitated brain homogenates from a typical MSA case. In contrast, PTA‐precipitated homogenates from the same brain regions of two G51D *SNCA* cases and amygdala from three DLB cases seeded only sparse aggregates with a punctate appearance (Figure 2).

### 3.5 Cryo‐EM Structures of α‐Synuclein Filaments

**FIGURE 3:**
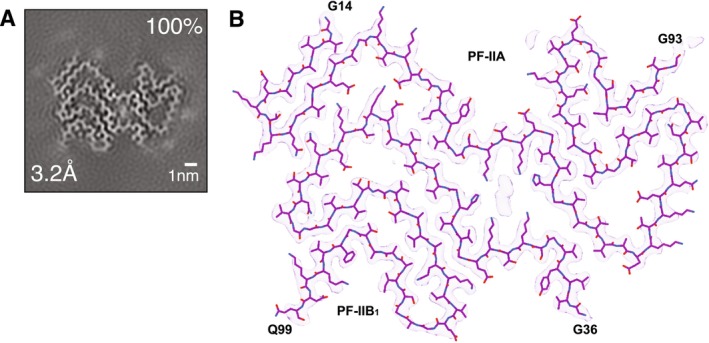
Cryo‐EM map and atomic model of Type II α‐synuclein filaments extracted from the frontal lobe of the FTLD‐synuclein case. (A). Cross‐section of α‐synuclein filaments through the cryo‐EM reconstruction, perpendicular to the helical axis and with a projected thickness of approximately one rung. The resolution (in Å) is indicated in the bottom left and the filament percentage in the top right. Scale bar, 1 nm. (B). Cryo‐EM density map of the Type II filament of α‐synuclein with overlaid atomic model. The protofilament cores extend from residues G14‐G93 and residues G36‐Q99 in PF‐IIA and PF‐IIB_1_, respectively.

The structure of α‐synuclein filaments from this case was determined to a resolution of 3.2 Å. It was identical to that of previously described MSA Type II filaments [2]. Filaments were each composed of two non‐identical protofilaments (IIA and IIB_1_), being made of an extended N‐terminal arm and a compact C‐terminal body, with the IIA protofilament consisting of residues G14‐G93 and the IIB_1_ protofilament being made of residues G36‐Q99 of α‐synuclein (Figure 3).

### 3.6 Genetics

**FIGURE 4:**
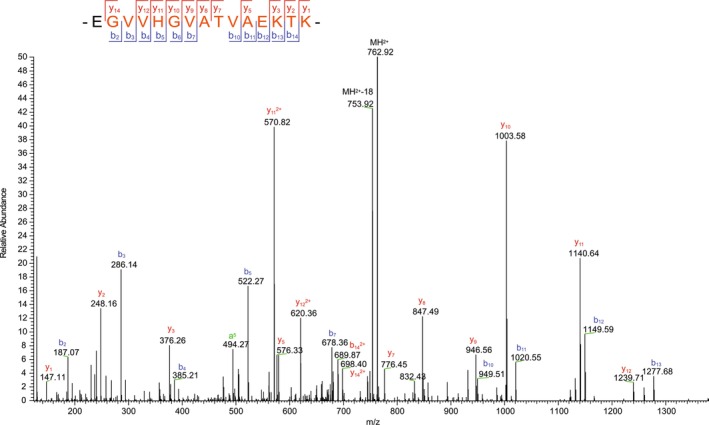
Identification of wild‐type α‐synuclein by mass spectrometry. The peptide sequence is shown at the top of each panel, together with collision‐induced fragmentation patterns; b ions (N‐terminal) and Y ions (C‐terminal) are generated during peptide fragmentation. Doubly charged ions are labelled “2+”. The ions that have a neutral loss are shown in green. Precursors are labelled MH; modified ions are labelled “‐18”. MS/MS spectrum of wild‐type α‐synuclein: EGVVHGVATVAEKTK.

No pathogenic variants were identified by WES. In particular, there were no single nucleotide variants in *SNCA*. Deletions and duplications in the coding region of *SNCA* were excluded by MLPA. The original labelling of this case as possibly carrying a G51D variant in *SNCA* was therefore ruled out. Mass spectrometry of the sarkosyl‐insoluble fractions used for cryo‐EM confirmed the presence of wild‐type α‐synuclein (Figure 4).

## 4 Discussion

MSA pathology is rarely associated with an FTD or a CBS phenotype [39]. While the frontotemporal distribution of pathology and heterogenous NCIs in the medial temporal lobe of the FTLD‐synuclein case described here raised the possibility of a G51D or an A53E variant in *SNCA*, no pathogenic *SNCA* variant was detected, and the morphological appearance of seeded inclusions was consistent with that of typical MSA. These findings, taken together with the presence of MSA Type II α‐synuclein filaments, indicate that FTLD‐synuclein is a rare pathological subtype of MSA. This is further supported by the observation of FTLD and globular Pick‐body‐like, ring‐shaped and NFT‐like inclusions in medial temporal structures of long‐duration MSA‐C and MSA‐P cases [14, 40, 41, 42, 43, 44]. In contrast to FTLD‐synuclein cases, these patients developed dementia/cortical features later in the disease course, suggesting that severe frontal and temporal cortical neuronal pathology can be a late feature of SND and OPCA. We previously reported a case of MSA‐MC with a CBS phenotype, suggesting that cortical neuronal dysfunction can also occur early in MSA in the absence of cortical atrophy [1].

The cryo‐EM structures of α‐synuclein filaments have previously been reported in five cases of typical MSA [2]. One case had only Type II filaments, one case had mostly Type I filaments and the other three cases had variable amounts of both filament types. It is yet to be determined whether Type I and Type II filaments are common to both neurones and glial cells, which may be of relevance for cases with a high burden of NCIs, such as the present case. The ratios of Type I to Type II filaments may also influence the disease phenotype, with Type II filaments being potentially associated with longer disease duration [2]. Here, we found only Type II MSA filaments. It thus appears that the structures of α‐synuclein filaments are the same in typical and atypical MSA. Consistent with previous experiments of seeded aggregation in rodents [45], PTA‐precipitated brain homogenates from G51D *SNCA* cases seeded sparse punctate inclusions in HEK293T cells that were similar to the results obtained using tissues from cases of DLB. These findings indicate that although some *SNCA* variant cases have neuropathological features overlapping MSA and Lewy body diseases [11, 12], the structures of α‐synuclein filaments from G51D *SNCA* cases may be more similar to the Lewy fold than the MSA folds.

Given the striking regional variation in MSA subtypes, it has been suggested that genetic susceptibility underlies the phenotypic variation in MSA. The patient's sister had a clinical history of MND, and there has been a previous report of MSA in a family with autosomal dominant MND and a hexanucleotide repeat expansion in *C9orf72* [46]. A subsequent study found pathogenic range *C9orf72* repeat expansions in two possible MSA cases [47]. However, TDP‐43 inclusions were not observed in the present case, consistent with an autopsy study of 100 MSA cases that did not identify pathogenic *C9orf72* repeat expansions [48]. Somatic *SNCA* variants are also potential contributors [49], but this has not been investigated in FTLD‐synuclein. Unlike typical MSA, where both sexes are affected equally [50], most of the previously reported FTLD‐synuclein and long‐duration MSA cases with severe frontotemporal cortical neuronal involvement have been women, suggesting that the female sex may be a factor in frontotemporal susceptibility to MSA pathology.

Although rare, FTLD‐synuclein cases expand the clinicopathological spectrum of both MSA and FTLD and have implications for our understanding of selective neuronal vulnerability in MSA and the interpretation of biomarker studies, such as those using α‐synuclein seed amplification assays and α‐synuclein positron emission tomography.

## Author Contributions

Patrick W. Cullinane, Sarah Wrigley, Eduardo de Pablo‐Fernández, Thomas T. Warner and Zane Jaunmuktane identified patients and collected clinical data. Zane Jaunmuktane, Janice Holton and Toby Curless. performed neuropathology assessments. Patrick W. Cullinane, Kirsten Ebanks and Amanda L. Woerman performed cell seeding experiments. Viorica Chelban, Yee Yen Goh and Henry Houlden performed whole exome sequencing. Sew Peak‐Chew and Catarina Franco performed mass spectrometry. Yang Yang, Sjors H.W. Scheres and Michel Goedert performed cryo‐EM. All authors contributed to the writing of the manuscript.

## Ethics Statement

Queen Square Brain Bank protocols and this study have been approved by the NHS Health Research Authority, Ethics Committee London‐Central (REC reference 23/LO/0044). All donors included in this study or their next of kin gave written informed consent at registration.

## Conflicts of Interest

The author Z.J. is an executive editor for Neuropathology and Applied Neurobiology. The Editors of Neuropathology and Applied Neurobiology are committed to peer‐review integrity and upholding the highest standards of review. As such, this article was peer‐reviewed by independent, anonymous expert referees, and the authors had no role in either the editorial decision or the handling of the paper. The authors declare that they have no other competing interests.

## Supporting information


**Figure S1** a, Cryo‐EM micrograph of filaments. Scale bar, 50 nm. b, 2D class average plots of filaments. Scale bar, 5 nm. c, Fourier shell correlation (FSC) curves for the cryo‐EM maps are shown in black; for the refined atomic model against the cryo‐EM map in red; for the atomic model refined in the first half map against that map in blue; for the refined atomic model in the first half map against the other half map in yellow.

**Table S1** Cryo‐EM data acquisition and structure determination.

## Data Availability

Cryo‐EM maps have been deposited in the Electron Microscopy Data Bank (EMDB) with the accession number EMD‐45979. Corresponding refined atomic models have been deposited in the Protein Data Bank (PDB) under accession number PDB:9CX6.
