# Supplementary figures and images for: Identical Seeding Characteristics and Cryo‐EM Filament Structures in FTLD‐Synuclein and Typical Multiple System Atrophy
Source: Neuropathol Appl Neurobiol. 2025 Mar 26;51(2):e70013. doi: 10.1111/nan.70013 (PMC11937994; doi:10.1111/nan.70013)

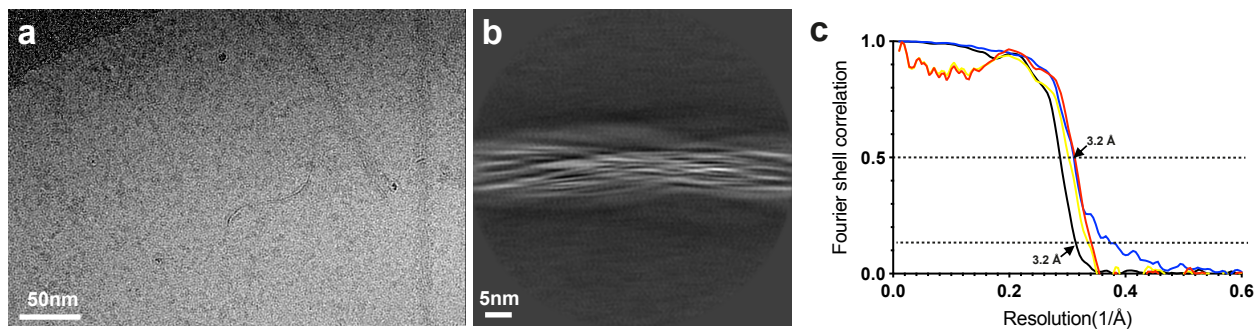

Supplement: Supplementary file 1 — Figure S1 a, Cryo‐EM micrograph of filaments. Scale bar, 50 nm. b, 2D class average plots of filaments. Scale bar, 5 nm. c, Fourier shell correlation (FSC) curves for the cryo‐EM maps are shown in black; for the refined atomic model against the cryo‐EM map in red; for the atomic model refined in the first half map against that map in blue; for the refined atomic model in the first half map against the other half map in yellow. [file NAN-51-e70013-s001.pdf]
