# Supplementary material for: Identical Seeding Characteristics and Cryo‐EM Filament Structures in FTLD‐Synuclein and Typical Multiple System Atrophy
Source: Neuropathol Appl Neurobiol. 2025 Mar 26;51(2):e70013. doi: 10.1111/nan.70013 (PMC11937994; doi:10.1111/nan.70013)
Supplement: Supplementary file 2 — Table S1 Cryo‐EM data acquisition and structure determination. [file NAN-51-e70013-s002.docx]

**Supplementary Table**

**Table S1. Cryo-EM data acquisition and structure determination.**

|  | FTLD case |
| --- | --- |
|  | (EMD-45979, PDB 9CX6) |
| **Data acquisition** |  |
| Electron gun | XFEG |
| Detector | K3 |
| Energy filter slit (eV) | 20 |
| Magnification | 105,000 |
| Voltage (kV) | 300 |
| Electron dose (e^–^/Å^2^) | 40 |
| Defocus range (μm) | 1.0 to 2.6 |
| Pixel size (Å) | 0.73 |
|  |  |
| **Map refinement** |  |
| Symmetry imposed | C1 |
| Initial particle images (no.) | 330,689 |
| Final particle images (no.) | 195,773 |
| Map resolution (Å) | 3.2 |
| FSC threshold | 0.143 |
| Helical twist (°) | -1.43 |
| Helical rise (Å) | 4.85 |
|  |  |
| **Model refinement** |  |
| Model resolution (Å) | 3.2 |
| FSC threshold | 0.5 |
| Map sharpening *B* factor (Å^2^) | -86 |
| **Model composition** |  |
| Non-hydrogen atoms | 4925 |
| Protein residues | 720 |
| Ligands | 0 |
| ***B* factors (Å^2^)** |  |
| Protein | 60 |
| **R.m.s. deviations** |  |
| Bond lengths (Å) | 0.0086 |
| Bond angles (°) | 1.5 |
| **Validation** |  |
| MolProbity score | 1.4 |
| Clashscore | 2.8 |
| Poor rotamers (%) | 0 |
| **Ramachandran plot** |  |
| Favored (%) | 95.0 |
| Allowed (%) | 5.0 |
| Disallowed (%) | 0 |

**Supplementary Figure Legend**

a, Cryo-EM micrograph of filaments. Scale bar, 50 nm.

b, 2D class average plots of filaments. Scale bar, 5 nm.

c, Fourier shell correlation (FSC) curves for the cryo-EM maps are shown in black; for the refined atomic model against the cryo-EM map in red; for the atomic model refined in the first half map against that map in blue; for the refined atomic model in the first half map against the other half map in yellow.
